# Deciphering the lipid–cancer nexus: comprehensive Mendelian randomization analysis of the associations between lipid profiles and digestive system cancer susceptibility

**DOI:** 10.1186/s12944-024-02191-0

**Published:** 2024-06-27

**Authors:** Yongyan Jin, Haiyan Zhou, Xiaoli Jin, Jun Wang

**Affiliations:** 1 Nursing Department, The Second Affiliated Hospital, Zhejiang University School of Medicine, Zhejiang, Hangzhou, 310000 Zhejiang China; 2 Department of Gastroenterology Surgery, The Second Affiliated Hospital, Zhejiang University School of Medicine, No.88 Jiefang Road, Hangzhou, 310000 Zhejiang China

**Keywords:** Digestive system cancers, Lipidomics, Mendelian randomization, Causal relationship

## Abstract

**Background:**

Digestive system cancers represent a significant global health challenge and are attributed to a combination of demographic and lifestyle changes. Lipidomics has emerged as a pivotal area in cancer research, suggesting that alterations in lipid metabolism are closely linked to cancer development. However, the causal relationship between specific lipid profiles and digestive system cancer risk remains unclear.

**Methods:**

Using a two-sample Mendelian randomization (MR) approach, we elucidated the causal relationships between lipidomic profiles and the risk of five types of digestive system cancer: stomach, liver, esophageal, pancreatic, and colorectal cancers. The aim of this study was to investigate the effect impact of developing lipid profiles on the risk of digestive system cancers utilizing data from public databases such as the GWAS Catalog and the UK Biobank. The inverse‒variance weighted (IVW) method and other strict MR methods were used to evaluate the potential causal links. In addition, we performed sensitivity analyses and reverse MR analyses to ensure the robustness of the results.

**Results:**

Significant causal relationships were identified between certain lipidomic traits and the risk of developing digestive system cancers. Elevated sphingomyelin (d40:1) levels were associated with a reduced risk of developing gastric cancer (odds ratio (OR) = 0.68, *P* < 0.001), while elevated levels of phosphatidylcholine (16:1_20:4) increased the risk of developing esophageal cancer (OR = 1.31, *P* = 0.02). Conversely, phosphatidylcholine (18:2_0:0) had a protective effect against colorectal cancer (OR = 0.86, *P* = 0.036). The bidirectional analysis did not suggest reverse causality between cancer risk and lipid levels. Strict MR methods demonstrated the robustness of the above causal relationships.

**Conclusion:**

Our findings underscore the significant causal relationships between specific lipidomic traits and the risk of developing various digestive system cancers, highlighting the potential of lipid profiles in informing cancer prevention and treatment strategies. These results reinforce the value of MR in unraveling complex lipid-cancer interactions, offering new avenues for research and clinical application.

**Supplementary Information:**

The online version contains supplementary material available at 10.1186/s12944-024-02191-0.

## Introduction

Digestive system cancers, including stomach, liver, esophageal, pancreatic, and colorectal cancers, pose a significant global health challenge, accounting for more than a quarter of all cancer cases [1]. The worldwide increase in the incidence of these cancers reflects not only demographic changes such as population growth and aging but also the spread of various risk factors to areas where they were not previously prevalent [2]. Among those factors, lifestyle factors, particularly dietary habits, have gained attention for their role in cancer development [3]. Socioeconomic development, which has led to a shift toward less healthy Western diets and increased consumption of processed foods, has been linked to an increasing prevalence of obesity and related metabolic disorders [4], which are associated with the increasing incidence of digestive system cancers. This background informed our study, the aim of which was to explore the intricate relationships between lipidomic profiles and the risk of developing digestive system cancers using Mendelian randomization (MR), offering a new perspective on potential prevention and treatment pathways.

Lipidomics has emerged as a significant area in cancer research, revealing that alterations in lipid metabolism are closely linked to cancer development and progression [5, 6]. Cancer cells exhibit changes in lipid synthesis, storage, and uptake, which are critical for membrane biogenesis and function and contribute to cancer cell survival in a changing microenvironment [7, 8]. The study of lipidomics offers insights into identifying novel biomarkers and therapeutic targets and developing lipid-inspired therapies. However, despite these advancements, the causal relationship between specific lipid profiles and cancer risk is not fully understood due to the inherent limitations associated with observational studies. This gap highlights the need for further research utilizing methods such as MR to clarify these relationships and explore the full potential of lipidomics in oncology.

Observational epidemiological studies, while invaluable for identifying potential risk factors for disease, are often limited by confounding and reverse causation. These limitations can compromise the reliability of studies investigatingthe causal effects of modifiable exposures on disease outcomes. MR has emerged as a powerful method to overcome these challenges, utilizing genetic variants as instrumental variables (IVs) [9]. By leveraging genetic variants that influence exposures of interest, MR enables researchers to generate evidence that is less prone to the biases that frequently affect the reliability of the results of observational studies [10]. This approach not only enhances the validity of causal inferences drawn from epidemiological data but also provides new paths for identifying interventions that could yield substantial health benefits [11]. The development of MR, including innovative variations such as bidirectional MR, has resulted in substantial expansion of its applicability and potential to inform public health and clinical practice.

Despite the acknowledged association between lipid profiles and digestive system cancers, existing research, which is primarily observational in nature, has yielded inconsistent findings regarding the causal nature of these relationships. Therefore, the aim of this study was to employ MR to investigate the causal relationships between lipidomic profiles and the risk of developing major digestive system cancers, including gastric, esophageal, colorectal, liver, and pancreatic cancers. Additionally, we sought to obtaina deeper understanding of the lipid–cancer nexus, paving the way for subsequent methodological discussions and analyses.

## Methods

### Study design

**Fig. 1:**
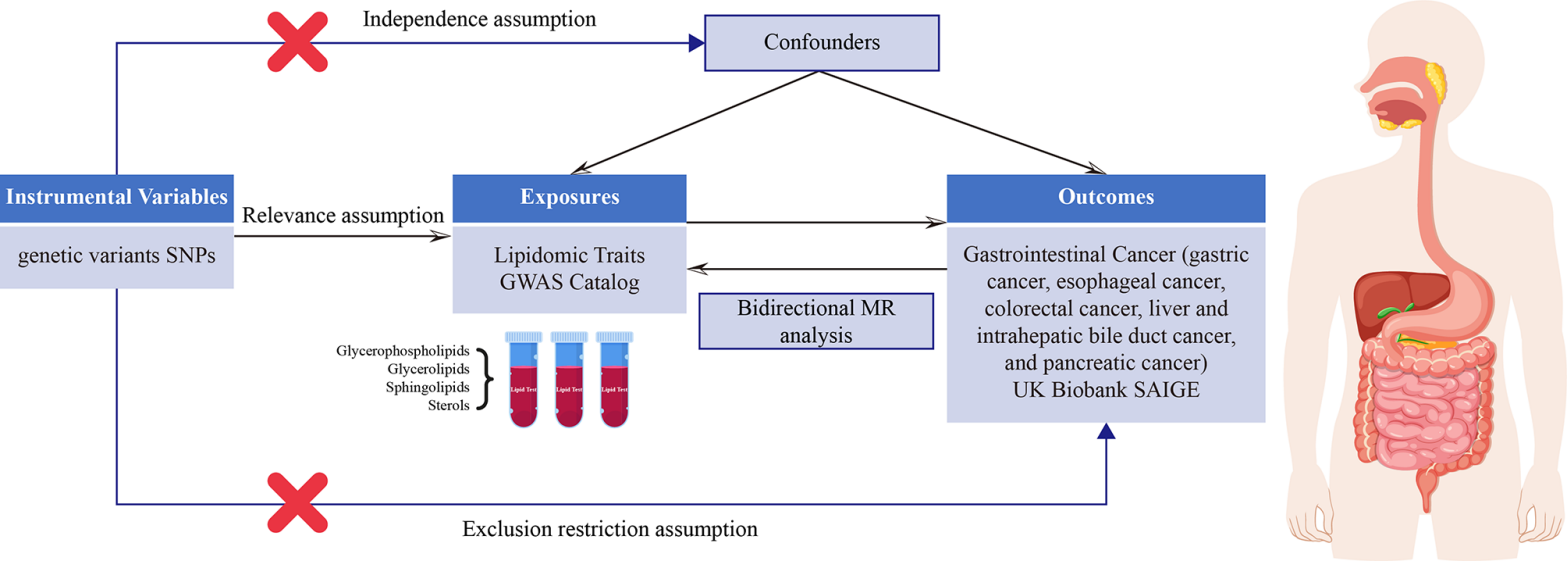
Diagram illustrating the conducted analyses

Our study employed a two-sample MR approach to investigate the causal relationship between lipidomic profiles and the risk of developing digestive system cancers. This design leverages genetic variants, specifically single-nucleotide polymorphisms (SNPs), associated with lipidomic traits as IVs. These SNPs were sourced from large-scale genome-wide association studies (GWASs) available in public databases such as the GWAS Catalog (https://www.ebi.ac.uk/gwas/). We utilized summary-level data, ensuring that the IVs were strongly associated with the exposure (lipidomic traits) but not directly associated with the outcome (digestive system cancer risk), thereby minimizing confounding. MR studies rely on three critical assumptions to ensure the validity of their causal inferences (Fig. 1). These assumptions are as follows: (1) Relevance assumption: This assumption states that the genetic variants must be robustly associated with the exposure of interest. (2) Independence assumption: This assumption posits that the genetic variants serving as IVs should be independent of confounders that affect both the exposure and the outcome. (3) Exclusion restriction assumption: This assumption states that the IVs affect the outcome only through the exposure and not through any alternative pathways. Meeting these assumptions is essential for the validity of MR studies.

### Data sources

In this MR analysis, we utilized two distinct data sources: genetic variants associated with lipidomic profiles and genetic variants associated with digestive system cancer outcomes. For lipidomic traits, we leveraged data from a GWAS of the plasma lipidome from the GWAS Catalog [12], which was published on October 31, 2023. This dataset provides comprehensive data on genetic variants associated with 179 kinds of lipid species (belonging to 13 lipid classes and 4 categories) in 7,174 Finnish individuals. Digestive system cancer outcome data, including data on gastric cancer, esophageal cancer, colorectal cancer, liver and intrahepatic bile duct cancer, and pancreatic cancer, were obtained from the UK Biobank SAIGE, a vast biomedical database and research resource comprising comprehensive, anonymized health and genetic data from 500,000 participants in the UK (https://pheweb.org/UKB-SAIGE/phenotypes). Detailed phenotypic information and genotypic data are shown in Table 1.

**Table 1:** Detailed phenotypic information and genotypic data of digestive system cancers

GWASID	Traits	SampleSize	Cases	Controls
ukb-saige-151	Cancer of stomach	393,926	554	393,372
ukb-saige-150	Cancer of esophagus	394,092	720	393,372
ukb-saige-153	Colorectal cancer	387,318	4562	382,756
ukb-saige-157	Pancreatic cancer	393,961	589	393,372
ukb-saige-155	Cancer of liver and intrahepatic bile duct	393,716	344	393,372

### Selection of IVs

We employed a multistep algorithm to select genetic variants from GWASs of the plasma lipidome in the GWAS Catalog. Initially, we identified SNPs significantly associated with key lipidomic traits (179 kinds of lipid species) using a threshold of *P* < 1 × 10^− 5^. This stringent significance level ensures a strong genetic instrument–exposure association. To mitigate the risk of pleiotropy, we excluded SNPs associated with confounding factors, which were identified using PhenoScanner V2 [13]. Furthermore, we calculated the F-statistics for each SNP to exclude weak IVs, setting a minimum threshold of 10 to ensure robustness [14]. The remaining SNPs were assessed for linkage disequilibrium (LD), combining those in high LD (r^2^ > 0.001) within a 10,000 kb window, ensuring independence among the IVs. In the bidirectional MR analysis, we designated digestive system cancers as the exposure variables, while lipidomic traits were treated as outcomes. The IVs were selected based on a significance threshold of *P* < 5 × 10^− 5^.

### Statistical analysis

We used the *TwoSampleMR* (v0.5.7) and *MendelianRandomization* (v0.9.0) R packages for our primary analysis. Initially, we used the inverse–variance weighted (IVW) [15] method as our primary analytic tool to estimate causal effects. Weighted median [16], MR–Egger [17], and weighted mode [18] methods were also used. In this study, several strict MR methods, including constrained maximum likelihood and model averaging-based MR (cML–MA) [19], contamination mixture (ConMix) [20], robust adjusted profile score (MR–RAPS), and the debiased inverse-variance weighted (dIVW) method, were used to estimate the direct effects of lipidomic traits on digestive system cancers. To assess potential pleiotropy and validate our findings, we conducted sensitivity analyses using the MR‒Egger and weighted median methods. The MR‒Egger approach tests for and corrects pleiotropic bias, providing an intercept term that indicates the presence of directional pleiotropy. We further used the Mendelian Randomization Pleiotropy RESidual Sum and Outlier (MR–PRESSO) [21] test to detect and correct for outliers, thereby ensuring that our results were not driven by individual SNPs with disproportionate influence. Finally, to assess the heterogeneity in our IV analysis, we used Cochran’s Q statistic [15]. We used an online website to perform the power calculation (https://sb452.shinyapps.io/power/), an online sample size and power calculator for MR with a binary outcome. To mitigate potential confounding effects stemming from the broader genetic association between lipids and digestive system cancers, we employed a linkage disequilibrium score regression (LDSC) analysis specifically focused on candidate lipids within our MR investigation [22, 23]. For multivariate MR (MVMR) analyses, we simultaneously assessed the effects of multiple significant lipids using the IVW method. All the statistical analyses were performed using R software (version 4.2.1), ensuring rigorous and reproducible results. All the statistical analyses were rigorously performed by employing two‒tailed tests, where a threshold of *P* < 0.05 was considered to indicate statistical significance.

## Results

### Causal effects of lipidomic traits on gastric cancer risk

**Fig. 2:**
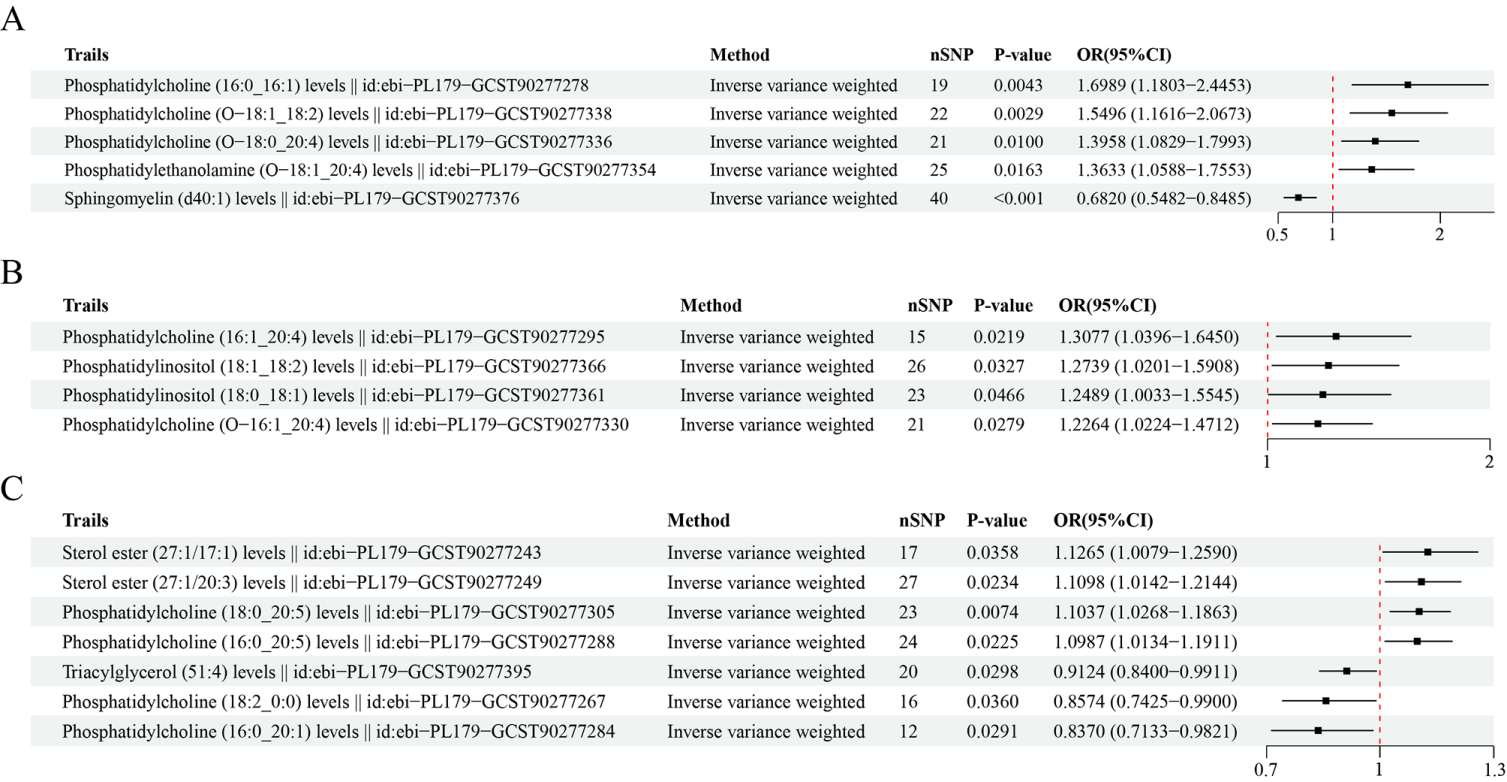
Forest plots of causal effect estimates of lipidomic traits on the risk of developing each type of digestive system cancer. **(A)** Gastric cancer. **(B)** Esophageal cancer. **(C)** Colorectal cancer

MR analysis revealed five significant causal relationships between lipidomic traits and the risk of developing gastric cancer (Fig. 2A). First, we identified a relationship between elevated levels of sphingomyelin (d40:1) and a decreased risk of developing gastric cancer (odds ratio (OR) = 0.68, 95% confidence interval (CI): 0.55–0.85, IVW_P < 0.001). In contrast, a lower phosphatidylethanolamine (O-18:1_20:4) level was associated with an increased risk of developing gastric cancer (OR = 1.36, 95% CI: 1.06–1.76; IVW_P = 0.02). Additionally, increased levels of phosphatidylcholine (16:0_16:1), phosphatidylcholine (O-18:0_20:4), and phosphatidylcholine (O-18:1_18:2) were associated with an increased risk of developing gastric cancer (OR = 1.70, 1.40, and 1.55, respectively; Supplementary Table 1). We adopted strict MR analysis algorithms, including cML–MA, ConMix, MR–RAPS, and dIVW, to estimate the direct effects of lipidomic traits on digestive system cancers. As shown in Table 2, all five causal effects of lipidomic traits on gastric cancer risk were strong. The robustness of our findings was confirmed through various sensitivity analyses, including Cochran’s Q test, MR-Egger regression, weighted median, and the leave-one-out approach.

**Table 2:** The results of strict MR analysis

Exposure	Outcome	Method	nSNP	Beta	SE	pval	OR
Phosphatidylcholine (16:0_16:1) levels || id: ebi-PL179-GCST90277278	Cancer of stomach || id: ukb-saige-151	Inverse variance weighted	19	0.530	0.186	0.004	1.699
		Contamination mixture method	19	0.896	0.268	0.004	2.449
		Robust adjusted profile score (RAPS)	19	0.604	0.194	0.002	1.829
		Debiased inverse-variance weighted method	19	0.555	0.191	0.004	1.742
		Constrained maximum likelihood	19	0.555	0.237	0.019	1.742
Phosphatidylcholine (O-18:0_20:4) levels || id: ebi-PL179-GCST90277336	Cancer of stomach || id: ukb-saige-151	Inverse variance weighted	21	0.333	0.130	0.010	1.396
		Contamination mixture method	21	0.285	0.148	0.066	1.330
		Robust adjusted profile score (RAPS)	21	0.318	0.138	0.021	1.374
		Debiased inverse-variance weighted method	21	0.343	0.134	0.010	1.409
		Constrained maximum likelihood	21	0.313	0.140	0.026	1.368
Phosphatidylcholine (O-18:1_18:2) levels || id: ebi-PL179-GCST90277338	Cancer of stomach || id: ukb-saige-151	Inverse variance weighted	22	0.438	0.147	0.003	1.550
		Contamination mixture method	22	0.638	0.186	0.001	1.893
		Robust adjusted profile score (RAPS)	22	0.457	0.163	0.005	1.580
		Debiased inverse-variance weighted method	22	0.459	0.156	0.003	1.583
		Constrained maximum likelihood	22	0.452	0.158	0.004	1.571
Phosphatidylethanolamine (O-18:1_20:4) levels || id: ebi-PL179-GCST90277354	Cancer of stomach || id: ukb-saige-151	Inverse variance weighted	25	0.310	0.129	0.016	1.363
		Contamination mixture method	25	0.402	0.184	0.088	1.495
		Robust adjusted profile score (RAPS)	25	0.310	0.139	0.025	1.364
		Debiased inverse-variance weighted method	25	0.321	0.134	0.017	1.378
		Constrained maximum likelihood	25	0.304	0.153	0.047	1.356
Sphingomyelin (d40:1) levels || id: ebi-PL179-GCST90277376	Cancer of stomach || id: ukb-saige-151	Inverse variance weighted	40	-0.383	0.111	0.001	0.682
		Contamination mixture method	40	-0.699	0.163	0.000	0.497
		Robust adjusted profile score (RAPS)	40	-0.437	0.120	0.000	0.646
		Debiased inverse-variance weighted method	40	-0.397	0.116	0.001	0.672
		Constrained maximum likelihood	40	-0.412	0.140	0.003	0.662

* SNP, single nucleotide polymorphism; SE, standard error; OR, odds ratio

Our bidirectional MR analysis did not reveal any evidence suggesting that gastric cancer risk causally influences the above lipidomic traits (Supplementary Table 1). This finding indicates that while lipid levels may influence the risk of developing gastric cancer, the presence of gastric cancer does not appear to causally alter lipid profiles within the studied population. A comprehensive MVMR analysis was utilized to examine the interrelationships among the five lipid traits. However, no significant associations were detected between the levels of these lipids and the risk of developing gastric cancer (*P* > 0.05, Supplementary Table 1).

### Causal effects of lipidomic traits on esophageal cancer risk

We observed a total of four significant causal effects of lipidomic traits on the risk of developing esophageal cancer (Fig. 2B). Increased phosphatidylcholine (16:1_20:4) levels were associated with a 30% increase in the risk of developing esophageal cancer (OR = 1.31, 95% CI: 1.04–1.65, *P* = 0.02). Higher levels of phosphatidylcholine (O-16:1_20:4) were also associated with an increased risk of developing esophageal cancer (OR = 1.23, 95% CI: 1.02–1.47; *P* = 0.03). In addition, both the phosphatidylinositol (18:0_18:1) and phosphatidylinositol (18:1_18:2) levels had positive causal effects on the risk of developing esophageal cancer (OR = 1.25 and 1.27, respectively, Supplementary Table 2). According to the results of strict MR analysis, most of the above four lipidomic traits still had direct effects on the risk of developing esophageal cancer (Supplementary Table 2). The application of Cochran’s Q test (*P* > 0.05), MR‒Egger intercept test (*P* > 0.05), and leave-one-out analysis (*P* > 0.05) revealed no signs of heterogeneity, directional pleiotropy, or issues with robustness in our MR analyses.

According to our bidirectional MR analysis, we did not find any evidence suggesting that esophageal cancer risk causally influences the above lipidomic traits (Supplementary Table 2). In addition, according to the LDSC and MVMR analyses, the levels of the above four lipids did not have significant causal relationships with the risk of developing esophageal cancer (*P* > 0.05, Supplementary Table 2).

### Causal effects of lipidomic traits on colorectal cancer risk

Utilizing genetic variants as IVs, we investigated seven potential causal relationships between lipid traits and the risk of developing colorectal cancer (Fig. 2C). Interestingly, the levels of phosphatidylcholine (18:2_0:0), phosphatidylcholine (16:0_20:1) and triacylglycerol (51:4) showed inverse relationships with colorectal cancer risk (Supplementary Table 3). An increase of 1 standard deviation in the phosphatidylcholine (18:2_0:0) level was associated with a 14% decrease in colorectal cancer risk (OR = 0.86, 95% CI: 0.74–0.99, *P* = 0.036). Higher phosphatidylcholine (16:0_20:1) levels were associated with a 16% reduction in the risk of developing colorectal cancer (OR = 0.84, 95% CI: 0.71–0.98, *P* = 0.029). Additionally, there was a slight negative correlation between triacylglycerol (51:4) levels and the risk of developing colorectal cancer (OR = 0.91, 95% CI: 0.84–0.99, *P* = 0.03). In contrast, the analysis indicated that two lipid traits were associated with an increased risk of developing colorectal cancer (OR = 1.13, *P* = 0.036 for sterol ester (27:1/17:1), OR = 1.11, *P* = 0.023 for sterol ester (27:1/20:3)). Higher levels of phosphatidylcholine (18:0_20:5) were associated with a slight (10%) increase in the risk of developing colorectal cancer (OR = 1.10, 95% CI: 1.03–1.19; *P* = 0.007). In the sensitivity analyses of all seven lipids, no heterogeneity or horizontal pleiotropy was detected (both Cochran’s Q and MR‒Egger test, *P* > 0.05). The leave-one-out analysis indicated that omitting any single SNP did not alter the our MR outcomes.Under a rigorous MR framework, the majority of the above seven lipid traits continued to demonstrate a direct impact on the risk of developing colorectal cancer (Supplementary Table 3). Conversely, there was no discernible evidence to suggest that the risk of developing colorectal cancer causally affects these lipidomic traits (Supplementary Table 3). LDSC revealed no significant genetic correlations between the above lipids and the risk of developing colorectal cancer (Supplementary Table 3). The results of the MVMR analysis showed that there were no potential causal relationships between the seven lipids and colorectal cancer (*P* > 0.05, Supplementary Table 3).

### Causal effects of lipidomic traits on liver and intrahepatic bile duct cancer risk

**Fig. 3:**
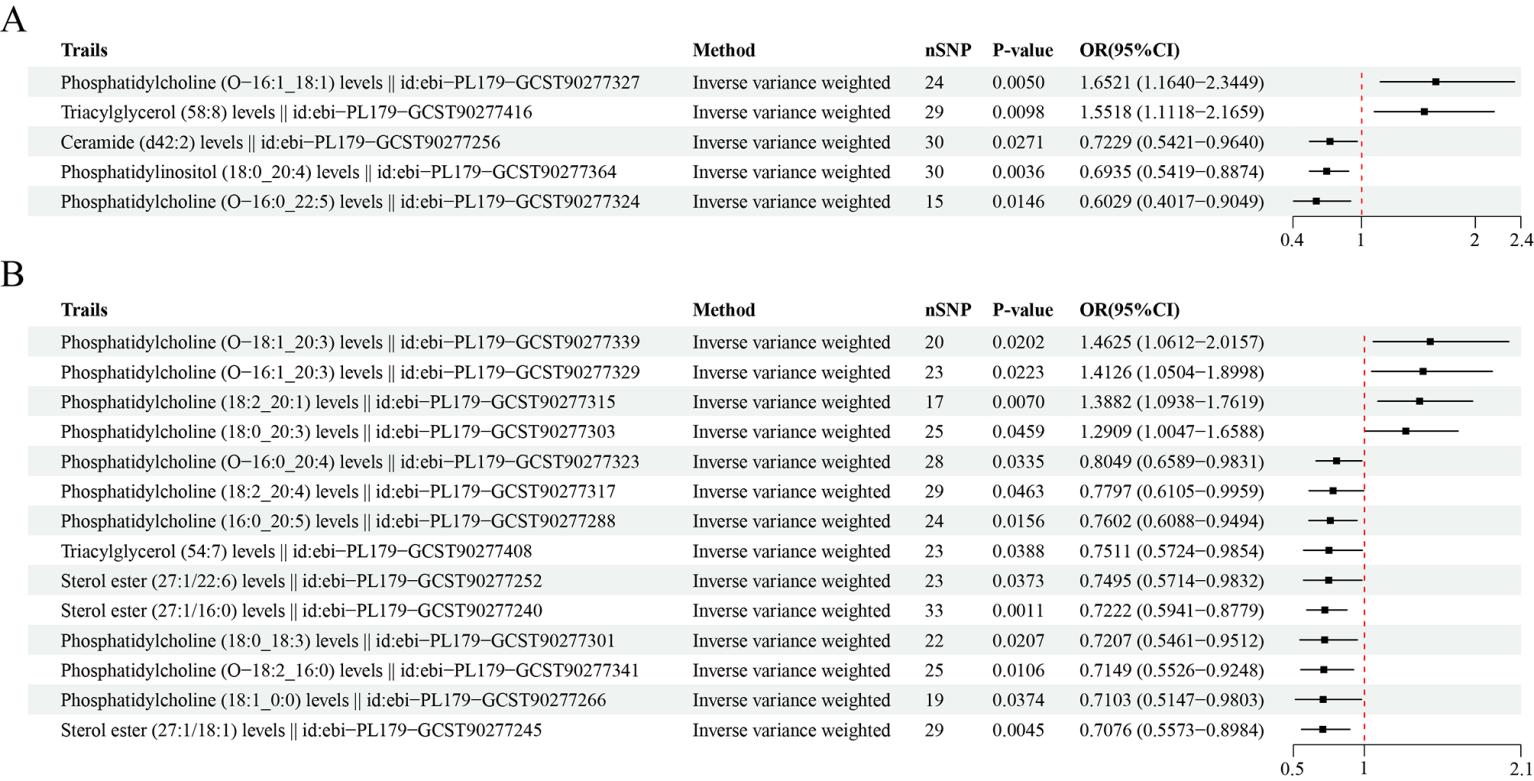
Forest plots of causal effect estimates of lipidomic traits on the risk of developing each type of digestive system cancer. **(A)** Liver and intrahepatic bile duct cancer. **(B)** Pancreatic cancer

Notably, increased levels of ceramide (d42:2) and phosphatidylinositol (18:0_20:4) and decreased levels of phosphatidylcholine (O-16:0_22:5) had protective effects against liver and intrahepatic bile duct cancer risk, as detailed in Table 3. An increase of one standard deviation in the level of ceramide (d42:2) was linked to a 28% decrease in the risk of developing colorectal cancer (OR = 0.72, 95% CI: 0.54–0.96, *P* = 0.027). Elevated levels of phosphatidylinositol (18:0_20:4) were correlated with a 31% lower risk of developing colorectal cancer (OR = 0.69, 95% CI: 0.54–0.89, *P* = 0.004). Additionally, a significant inverse relationship was observed between the level of phosphatidylcholine (O-16:0_22:5) and liver cancer risk (OR = 0.60, 95% CI: 0.40–0.90, *P* = 0.015). Conversely, our findings indicated that increased liver cancer risk was associated with two lipid traits (OR = 1.65, *P* = 0.005 for phosphatidylcholine (O-16:1_18:1), OR = 1.55, *P* = 0.01 for triacylglycerol (58:8); Fig. 3A). When performing sensitivity analyses, neither Cochran’s Q test or the MR–Egger test indicated any heterogeneity and horizontal pleiotropy. Additionally, the MR–PRESSO test revealed no evidence of pleiotropy, validating the accuracy of our results.

**Table 3:** Causal effect of lipidomic traits on liver and intrahepatic bile duct cancer risks using various MR methods

Exposure	Method	nSNP	Beta	SE	pvalue	OR	OR_lci95	OR_uci95	p_FDR
Ceramide (d42:2) levels || id: ebi-PL179-GCST90277256	Inverse variance weighted	30	-0.324	0.147	0.027	0.723	0.542	0.964	0.027
	Contamination mixture method	30	-0.658	0.265	0.061	0.518	0.308	0.871	0.061
	Robust adjusted profile score (RAPS)	30	-0.348	0.157	0.027	0.706	0.519	0.961	0.027
	Debiased inverse-variance weighted method	30	-0.334	0.152	0.028	0.716	0.532	0.964	0.028
	Constrained maximum likelihood	30	-0.338	0.193	0.079	0.713	0.489	1.040	0.079
Phosphatidylcholine (O-16:0_22:5) levels || id: ebi-PL179-GCST90277324	Inverse variance weighted	15	-0.506	0.207	0.015	0.603	0.402	0.905	0.015
	Contamination mixture method	15	-1.174	0.602	0.705	0.309	0.095	1.006	0.705
	Robust adjusted profile score (RAPS)	15	-0.741	0.276	0.007	0.477	0.277	0.819	0.007
	Debiased inverse-variance weighted method	15	-0.528	0.210	0.012	0.590	0.391	0.889	0.012
	Constrained maximum likelihood	15	-0.436	0.260	0.093	0.646	0.389	1.075	0.093
Phosphatidylcholine (O-16:1_18:1) levels || id: ebi-PL179-GCST90277327	Inverse variance weighted	24	0.502	0.179	0.005	1.652	1.164	2.345	0.005
	Contamination mixture method	24	0.786	0.273	0.019	2.194	1.285	3.746	0.019
	Robust adjusted profile score (RAPS)	24	0.526	0.192	0.006	1.693	1.161	2.468	0.006
	Debiased inverse-variance weighted method	24	0.524	0.189	0.005	1.689	1.167	2.444	0.005
	Constrained maximum likelihood	24	0.474	0.219	0.030	1.606	1.046	2.465	0.030
Phosphatidylinositol (18:0_20:4) levels || id: ebi-PL179-GCST90277364	Inverse variance weighted	30	-0.366	0.126	0.004	0.693	0.542	0.887	0.004
	Contamination mixture method	30	-0.581	0.186	0.003	0.559	0.388	0.805	0.003
	Robust adjusted profile score (RAPS)	30	-0.382	0.133	0.004	0.682	0.526	0.885	0.004
	Debiased inverse-variance weighted method	30	-0.375	0.129	0.004	0.688	0.534	0.886	0.004
	Constrained maximum likelihood	30	-0.375	0.144	0.009	0.687	0.518	0.910	0.009
Triacylglycerol (58:8) levels || id: ebi-PL179-GCST90277416	Inverse variance weighted	29	0.439	0.170	0.010	1.552	1.112	2.166	0.010
	Contamination mixture method	29	0.966	0.309	0.011	2.627	1.434	4.810	0.011
	Robust adjusted profile score (RAPS)	29	0.447	0.240	0.063	1.563	0.976	2.505	0.063
	Debiased inverse-variance weighted method	29	0.460	0.175	0.008	1.584	1.125	2.230	0.008
	Constrained maximum likelihood	29	0.437	0.209	0.036	1.548	1.028	2.331	0.036

* SNP, single nucleotide polymorphism; SE, standard error; OR, odds ratio; CI, confidence interval; FDR, false discovery rate

Using multiple stringent MR methods, most of the five lipid traits studied were shown to have a consistent influence on liver cancer risk, as reported in Table 3. Importantly, there was no reverse-causal effect of liver cancer on these lipidomic profiles, as shown in Supplementary Table 4. LDSC analysis revealed no significant genetic correlations between the levels of the above lipids and the risk of developing liver cancer (Supplementary Table 4). In the MVMR analysis, we only explored the causal relationship between phosphatidylinositol (18:0_20:4) levels and liver and intrahepatic bile duct cancer risk (OR = 0.64, 95% CI: 0.42–0.96, *P* = 0.03; Supplementary Table 4).

### Causal effects of Lipidomic traits on Pancreatic Cancer Risk

Similarly, a total of 14 causal relationships were observed between lipidomic traits and the risk of developing pancreatic cancer based on the IVW method (Fig. 3B, Supplementary Table 5). Among these lipidomic traits, increased levels of phosphatidylcholine (O-18:1_20:3) were associated with an increased risk of developing pancreatic cancer (OR = 1.46, 95% CI: 1.06–2.02; *P* = 0.02). In contrast, sterol ester (27:1/18:1) levels were found to have an inverse association with the risk of developing pancreatic cancer. Higher sterol ester (27:1/18:1) levels were associated with a 29% reduction in the risk of developing esophageal cancer (OR = 0.71, 95% CI: 0.56–0.90, *P* = 0.005). Our use of Cochran’s Q test and MR‒Egger test indicated that our MR analyses were free from heterogeneity and directional pleiotropy, indicating robustness of the results. Furthermore, the leave-one-out analysis revealed that no individual SNP significantly influenced the MR estimates.

**Fig. 4:**
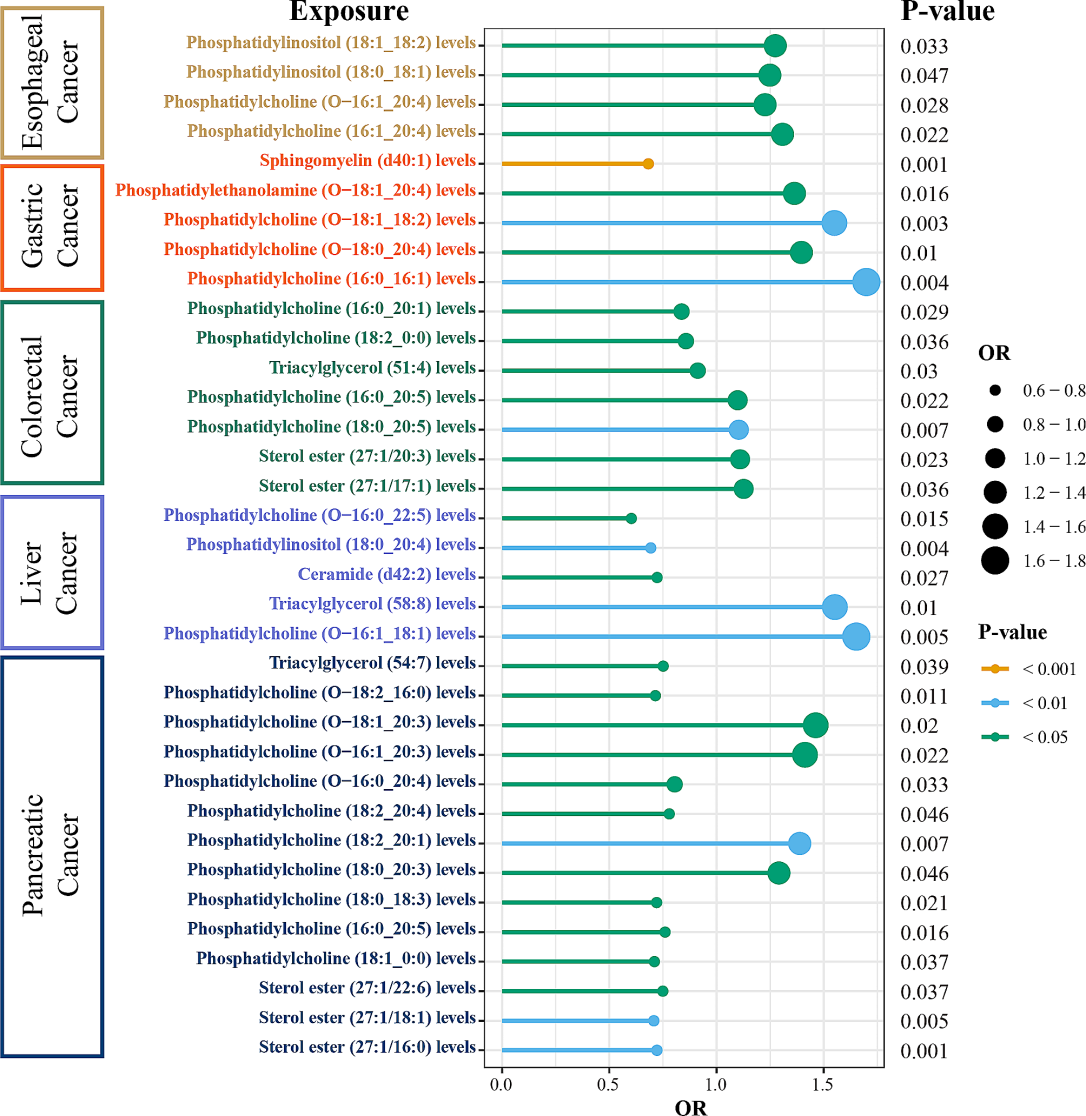
Pooled positive results among five digestive system cancers

According to strict MR analysis algorithms, including cML‒MA, ConMix, MR‒RAPS, and dIVW, most of the above lipidomic traits still had direct effects on the risk of developing pancreatic cancer (Supplementary Table 5). However, the levels of phosphatidylcholine (18:0_18:3), phosphatidylcholine (18:1_0:0), and triacylglycerol (54:7) were not significantly different. Finally, the investigation of pancreatic cancer revealed no reverse‒causal effect of pancreatic cancer risk on the above lipidomic traits (Supplementary Table 5). In Fig. 4, we pooled all the positive results among digestive system cancers. LDSC revealed no significant genetic correlations apart from sterol ester (27:1/18:1) levels (r_g_ = 0.936, *P* = 0.02, Supplementary Table 5). According to the MVMR analysis, sterol ester (27:1/18:1) levels and phosphatidylcholine (16:0_20:5) levels remained causally related to pancreatic cancer risk (Supplementary Table 5).

## Discussion

In our study, we sought to elucidate the causal relationships between lipidomic profiles and the risk of developing various digestive system cancers using MR. This body of work highlights the diverse effects of lipid traits on cancer risk, underscoring the complexity of lipid-cancer interactions and the potential for targeted preventive strategies.

Cancer cells are known to alter their metabolic pathways to sustain their malignancy. Aberrant lipid metabolism is closely associated with various cancers. Cancer cells utilize lipid metabolism for energy, membrane components, and signaling molecules [24]. An abundance of lipids may enhance the ability of tumor cells to develop, colonize, and spread [25]. These adjustments include modifications to the composition of the lipid membrane to facilitate invasion into different environments and to navigate mechanisms of cell death, in addition to enhancing lipid breakdown and synthesis for the generation of energy and protection against oxidative stress. Additionally, key genes and proteins associated with fat metabolism are considered potential markers for predicting outcomes in different cancers and are related to patient survival or the likelihood of cancer recurrence [26].

Notably, in our study, we found an association between elevated levels of sphingomyelin (d40:1) and reduced gastric cancer risk (OR = 0.682). The relationship between sphingomyelin and the risk of developing gastric cancer has been explored in various studies, revealing significant insights into the potential role of sphingomyelin in cancer development and progression. Compared with adjacent noncancerous tissues, gastric cancer samples exhibited higher levels of sphingomyelin [27]. One study revealed that sphingomyelin (d18:0/18:1(9Z)) was more abundant in patients with early gastric cancer than in healthy control participants, suggesting its potential as a biomarker for the early diagnosis of gastric cancer [28]. Three subclasses of phosphatidylcholine were positively associated with the risk of developing gastric cancer. A study revealed that serum phospholipids, including phosphatidylcholine, were more abundant in patients with early gastric cancer than in healthy controls, suggesting a role for these phospholipids in cancer development and as indicators for early detection [29]​.

We also observed that increased phosphatidylcholine and phosphatidylinositol levels were associated with increased esophageal cancer risk. A study using liquid chromatography-quadrupole time-of-flight mass spectrometry revealed dysregulation of phosphatidylcholines, indicating potential perturbations in phosphocholine metabolism specific to esophageal cancer [30]. However, there have been no studies on the role of phosphatidylinositol in the development of esophageal cancer. In colorectal cancer, higher sterol ester and phosphatidylcholine levels were associated with a slight increase in risk. Sterol metabolism plays a significant role in cellular processes, and alterations in this metabolic pathway have been associated with various types of cancer [31].

Notably, we identified a total of 14 different lipid phenotypes associated with the risk of developing pancreatic cancer. Recent studies have proven the crucial role of lipid metabolism in pancreatic cancer development [32]. Mounting research indicates that the progression and treatment resistance of pancreatic cancer can be fueled by lipid metabolism via the augmentation of lipid synthesis, accumulation, and breakdown [33]. According to our results, a total of four subclasses of phosphatidylcholine were positively related to the risk of developing pancreatic cancer. However, six subclasses of phosphatidylcholine were inversely related to risk, suggesting bidirectional roles.

The MR approach employed in our study is distinguished by its usefulness for assessing causal relationships, circumventing many of the limitations inherent in observational studies, such as confounding and reverse causality. This method leverages genetic variants as IVs to infer causation, assuming that these variants are associated with the exposure but not with any confounders of the outcome, making it a powerful tool for causal inference in epidemiology [34]. However, there are potential limitations to the MR approach that must be acknowledged. One significant limitation is the reliance on summary-level data from different sources, which might not always accurately capture individual-level variability and interactions. Meanwhile, the sample size of the GWAS data is one of our limitations. We need to further increase the sample size to enhance the reliability of the results. In our study, when selecting *P* < 5 × 10^− 8^ did not include enough SNPs for analysis, it may lead to low statistical power and issues with weak instrument variables. Thus, we chose *P* < 1 × 10^− 5^ as the threshold because we wanted to include more SNPs potentially related to lipid metabolism. So our results need to be interpreted with more caution. Additionally, while the use of MR can significantly reduce the confounding observed with the use of other methods, it cannot eliminate it entirely, particularly if there are unmeasured confounders that affect both the genetic instruments and the outcome. Another challenge is the generalizability of the results across different populations, as the genetic variants used as IVs might have different effects on different ethnic groups, especially the East Asian population, potentially limiting the applicability of the findings to broader populations.

Thus, when comparing and contrasting the findings of this study with those of previous studies in the field of lipidomics and cancer, we not only used conventional MR analysis methods (IVW, weighted median, MR‒Egger, and weighted mode) but also used a variety of rigorous MR algorithms, including cML–MA, ConMix, MR–RAPS, and dIVW. ConMix helps identify groups of genetic variants with similar causal estimates, which may represent different mechanisms through which risk factors influence outcomes. Additionally, it performs robustly and effectively in the presence of invalid IVs, achieving the lowest mean squared error compared to other robust methods across a range of realistic scenarios [20]. However, because of negative heritability, the results of LDSC analysis for certain lipids and cancer may exhibit instability. This may lead to weak causal relationships and requires validation in a larger cohort [35, 36].

The findings from our study underscore the critical role of lipidomic profiles in understanding cancer risk, particularly in digestive system cancers. This necessitates further exploration into how specific lipid species contribute to cancer development. First, we should perform lipidomics, including the use of peripheral blood and tissue samples from patients, to validate the above results. Future research should also focus on functional studies and experimental validation to elucidate the underlying mechanisms involved. Moreover, we will also focus on the impact of lipidomic profiles on specific subtypes of digestive system cancers, including molecular subtyping, pathological subtyping, or immunological subtyping. Different subtypes of cancer, such as lung cancer, may exhibit distinct lipidomic metabolic characteristics [37, 38]. The potential of integrating lipidomic profiles into clinical practice is promising. By incorporating these profiles into cancer risk assessment and prevention strategies, we can move toward more personalized and targeted therapeutic approaches, opening new pathways in the fight against cancer.

## Conclusion

In conclusion, our study significantly advances the understanding of the complex relationship between lipid metabolism and the development of digestive system cancers. By highlighting specific lipidomic traits associated with increased or decreased cancer risk, we highlight the potential of lipidomic modulation as a pioneering approach for cancer prevention and treatment. These findings emphasize the critical need for continued research in this promising field, suggesting that targeting lipid metabolism could open new avenues in the fight against cancer.

### Electronic supplementary material

Below is the link to the electronic supplementary material.

Supplementary Material 1: Table 1. Results of the main MR, bidirectional MR and MVMR analyses in gastric cancer.

Supplementary Material 2: Table 2. Results of the main MR, strict MR, bidirectional MR, LDSC and MVMR analyses in esophageal cancer patients.

Supplementary Material 3: Table 3. Results of the main MR, strict MR, bidirectional MR, LDSC and MVMR analyses in colorectal cancer.

Supplementary Material 4: Table 4. Results of the main MR, bidirectional MR, LDSC and MVMR analyses in liver cancer.

Supplementary Material 5: Table 5. Results of the main MR, strict MR, bidirectional MR, LDSC and MVMR analyses in pancreatic cancer.

## Data Availability

No datasets were generated or analysed during the current study.
